# Impact of an integrated community-based model of care for older people with complex conditions on hospital emergency presentations and admissions: a step-wedged cluster randomized trial

**DOI:** 10.1186/s12913-021-06668-x

**Published:** 2021-07-16

**Authors:** Jennifer Mann, Fintan Thompson, Robyn McDermott, A. Esterman, Edward Strivens

**Affiliations:** 1 Cairns and Hinterland Hospital and Health Service, PO Box 906, Cairns, Qld 4870 Australia; 2 College of Public Health, Medicine and Veterinary Sciences, James Cook University, Townsville, Qld 4811 Australia; 3 Clinical and Health Sciences, University of South Australia, GPO Box 2471, Adelaide, SA 5001 Australia; 4 College of Medicine and Dentistry, James Cook University, Townsville, Qld 4811 Australia

**Keywords:** Older person, Emergency department, Hospital admissions, Integration, Primary care, Complexity

## Abstract

**Background:**

Health systems must reorient towards preventative and co-ordinated care to reduce hospital demand and achieve positive and fiscally responsible outcomes for older persons with complex needs. Integrated care models can improve outcomes by aligning primary practice with the specialist health and social services required to manage complex needs. This paper describes the impact of a community-facing program that integrates care at the primary-secondary interface on the rate of Emergency Department (ED) presentation and hospital admissions among older people with complex needs.

**Methods:**

The Older Persons Enablement and Rehabilitation for Complex Health Conditions (OPEN ARCH) study is a multicentre randomised controlled trial with a stepped wedge cluster design. General practitioners (GPs; *n* = 14) in primary practice within the Cairns region are considered ‘clusters’ each comprising a mixed number of participants. 80 community-dwelling persons over 70 years of age if non-Indigenous and over 50 years of age if Indigenous were included at baseline with no new participants added during the study. Clusters were randomly assigned to one of three steps that represent the time at which they would commence the OPEN ARCH intervention, and the subsequent intervention duration (3, 6, or 9 months). Each participant was its own control. GPs and participants were not blinded. The primary outcomes were ED presentations and hospital admissions. Data were collected from Queensland Health Casemix data and analysed with multilevel mixed-effects Poisson regression modelling to estimate the effectiveness of the OPEN ARCH intervention. Data were analysed at the cluster and participant levels.

**Results:**

Five clusters were randomised to steps 1 and 2, and 4 clusters randomised to step 3. All clusters (*n* = 14) completed the trial accounting for 80 participants. An effect size of 9% in service use (95% CI) was expected. The OPEN ARCH intervention was found to not make a statistically significant difference to ED presentations or admissions. However, a stabilising of ED presentations and a trend toward lower hospitalisation rates over time was observed.

**Conclusions:**

While this study detected no statistically significant change in ED presentations or hospital admissions, a plateauing of ED presentation and admission rates is a clinically significant finding for older persons with complex needs. Multi-sectoral integrated programs of care require an adequate preparation period and sufficient duration of intervention for effectiveness to be measured.

**Trial registration:**

The OPEN ARCH study received ethical approval from the Far North Queensland Human Research Ethics Committee, HREC/17/QCH/104–1174 and is registered on the Australian and New Zealand Trials Registry, ACTRN12617000198325p.

**Supplementary Information:**

The online version contains supplementary material available at 10.1186/s12913-021-06668-x.

## Introduction

### Background

Health systems are under increasing pressure to reduce potentially preventable hospital demand [1, 2]. An ageing population and rise in multi-morbidity increase hospital use and acuity of presentation, particularly amongst the older person with complex needs [1, 2]. Complexity arises from the interface between medical diagnosis (multi-morbidity, frailty, and geriatric conditions) and personal contextual dynamics (socioeconomic status, culture, and environment) [3, 4]. Complexity increases vulnerability to functional decline and increases the likelihood that an older person will require hospital care [1, 2, 4].

Health systems must reorient towards preventative and coordinated care to reduce hospital demand and achieve positive and fiscally responsible client outcomes [2, 5]. Preventative care is best delivered in the community by the primary contact physician [2, 5]. However, multi-morbidity, geriatric syndromes, and psychosocial complexity are often challenging for the General Practitioner to manage in isolation [6, 7].

Integrated care models can improve outcomes for the older person by aligning primary practice with the specialist health care and social services required to manage complex needs [8, 9]. This approach views the General Practitioner (GP) as the central integrating function whereby care continuity is maintained by primary practice and the needs of the individual are addressed comprehensively by an integrated team of collaborators [10].

Integrated approaches to care for the older person have an established international history. TeWhiringa Ora is a broadly cited community-facing model that has shown improved access to health and social care to reduce hospital admission and length of stay in New Zealand [11, 12]. In Australia, the Hospital Admission Risk Program (HARP), and Health-One Mt Druitt, also report a decrease in the number of Emergency Department (ED) presentations amongst participants with the success of these various models attributed to improved access to primary and specialist health care and community-based social supports [13, 14].

In 2016, The Queensland Department of Health released the Integrated Care Innovation Fund (ICIF) to promote integration between primary care and specialist hospital services [15]. The Older Persons Enablement and Rehabilitation for Complex Health Conditions (OPEN ARCH) program was developed in Far North Queensland under this funding arrangement and delivered via a partnership between the Cairns and Hinterland Hospital and Health Service and the North Queensland Primary Health Network. This paper describes the impact of the OPEN ARCH program on the rate of ED presentations and hospital admissions among OPEN ARCH study participants.

## Methods

Before the OPEN ARCH intervention was introduced, participants had access to routine GP care. This included GP access to the online referral pathway for aged care supports (My Aged Care), and GP referral for community allied health and nursing interventions.

### The OPEN ARCH intervention

**Fig. 1:**
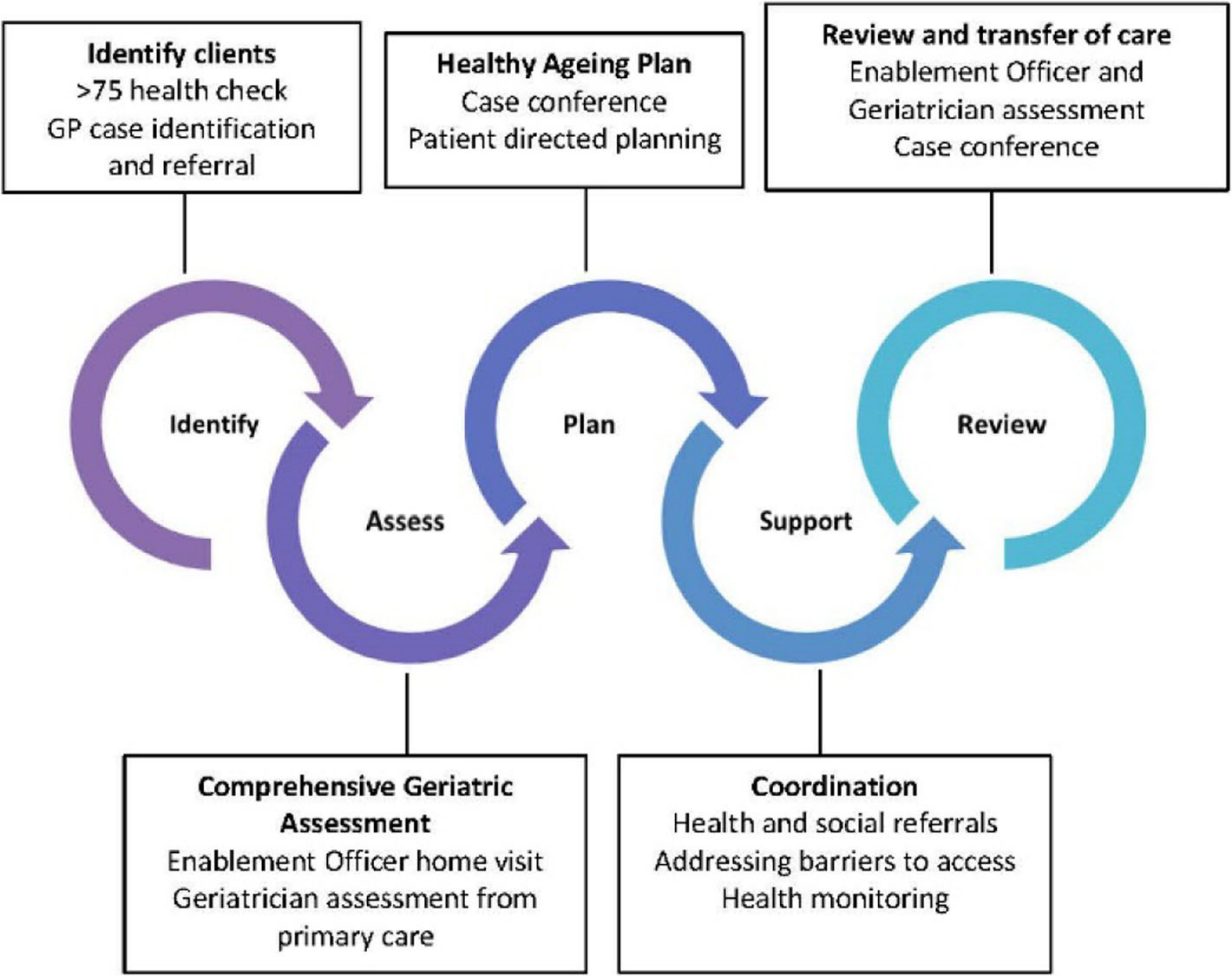
Service flow of the OPEN ARCH intervention [16].

OPEN ARCH (Older Persons Enablement and Rehabilitation for Complex Health Conditions), provides comprehensive geriatric assessment and client enablement for community-dwelling older persons with complex needs. The intervention is delivered in the primary care setting and features a collaboration between the client, treating GP, geriatric specialist and enablement officer (clinical nurse). Service flow is illustrated in Fig. 1 [16]. The OPEN ARCH model of care, study design, recruitment, participants, and data collection methods are described in detail elsewhere and summarised in the remainder of this methods section [16, 17]. The OPEN ARCH intervention was maintained for each participant from their intervention commencement date, through to the completion of the study unless otherwise indicated in the Results section, below.

### Trial design

**Fig. 2:**
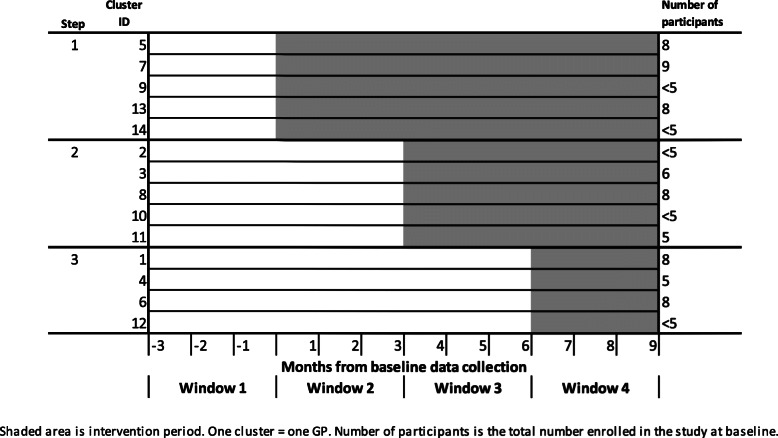
Roll-out diagram for the OPEN ARCH stepped wedge randomised controlled trial

The OPEN ARCH study is a multicentre randomised controlled trial with a stepped wedge cluster design. The stepped wedge design allows all participants to receive the treatment intervention, which is the preferred methodology for studies in which the intervention is predicted to do more good than harm, allows participants to act as their own control and detect trends and changes associated with time [18]. A stepped wedge trial has random and sequential crossover of clusters from control to intervention. At the first step, all of the clusters are in the control group, whereas at the end of the final step, all clusters are in the intervention group. In our study, General practitioners (GPs) were the clusters, each contributing 1–9 participants (clients) to the study. All participants commenced the study at baseline with no further participants added during the study period. These were randomised at baseline to one of three intervention steps using Excel’s randomization function (Fig. 2). The step to which each cluster was assigned determined the start date of the intervention for that cluster, with three-months between the commencement of each step. Step One included 5 clusters with a three-month control period and 9 months of intervention, Step Two included 5 clusters and a six-month control period and 6 months intervention, and Step Three included four clusters with a control period of 9 months followed by three-months of intervention (Fig. 2).

The OPEN ARCH study received ethics approval from the Far North Queensland Human Research Ethics Committee, HREC/17/QCH/104–1174 and is registered on the Australian and New Zealand Trials Registry, ACTRN12617000198325p. Detail of the trial design is provided in Kinchin et al. 2018 [17].

### Participants

Community-dwelling persons over 70 years of age if non-Indigenous and over 50 years of age if Aboriginal and/or Torres Strait Islander (Indigenous) were eligible for OPEN ARCH. The lower age requirement for Indigenous participants aligns with Commonwealth Government recognition of the specific health needs of Indigenous persons and the associated eligibility threshold for aged care services [19]. Older persons were not eligible if they were under the care of a geriatrician, receiving a program of co-ordinated care (such as transition care program or nurse navigation), or had a cognitive deficit and no substitute decision-maker.

### Recruitment

General Practitioners provided the researchers with an anonymised list of those older persons in their care whom they determined through routine clinical assessment as having complex needs. Using simple random selection, one researcher (JM) selected up to 12 older persons from each GP who were then approached by their GP and provided oral consent to be contacted by the OPEN ARCH team. The Participant Information Form was provided to the participants during a face-to-face meeting with an OPEN ARCH team member (including researcher JM) before the provision of informed written consent. Participant recruitment was completed over 7 weeks.

### Setting

The OPEN ARCH study was conducted with 14 GPs from 5 GP clinics in the Cairns and Hinterland region. Two GP clinics were an Aboriginal and Community Controlled Health Organisation. Cairns is located in Far North Queensland, Australia. The proportion of the Cairns population aged over 65 years is greater than the State average and Aboriginal and Torres Strait Islander persons comprise 14% of the population (compared with 4% across the State) [20]. In 2014/215 the Cairns region had the highest rate of potentially preventable hospital admissions in Queensland [19].

### Outcome measures

The number of ED presentations and hospital admissions at the local public health service for each participant was provided by the Cairns and Hinterland Hospital and Health Service from routinely collected health service data within the Casemix data collection system [21]. The time periods for these data comprised 3 months prior to each individual’s baseline collection of study measures (i.e., Window 1) and successive three-month periods (i.e., Windows 2–4) before each subsequent collection of study measures. For admitted patient data, admissions for blood transfusions and renal dialysis were excluded. Inpatient stays that involved a transfer between wards were combined to create a single episode of care.

Potentially Preventable Hospitalisations (PPHs) were flagged based on primary and secondary International Statistical Classification of Diseases and Related Health Problems, Tenth Revision, Australian Modification (ICD-10-AM) codes [22]. A PPH was identified if the ICD-10-AM codes met the criteria defined in the Australian Institute of Health and Welfare National Healthcare Agreement: PI 18–Selected potentially preventable hospitalisations, 2018 [23]. The PPHs were broadly categorised as Vaccine-preventable, Chronic, or Acute, although subcategories were also created (e.g., Pneumonia and influenza, vaccine preventable).

### Sample size

This study aimed for a sample size of 120 participants, to provide 80% power and detect a 9% difference (effect size) in service use with statistical significance at the 5% level. The effect size was based on change in health service use reported in a similar study by Bird et al [13]. An estimate of 120 participants was determined to allow for 25% censoring and a practical recruitment rate for each GP (clusters), which was considered 10–12 participants. This sample size calculation procedure is described in detail in the OEPN ARCH study protocol and by Kinchin et al [12, 17].

Of the participants identified by their GP as meeting eligibility criteria, 92 were randomly selected and invited to participate. Following enrolment, 12 participants were removed from the study due to withdrawing consent (*n* = 7), commencing support through a separate enhanced care service (*n* = 4) and, changing GP (*n* = 1). A total of 80 participants commenced the OPEN ARCH study. While this represented a censoring rate lower than anticipated (i.e., 13% compared to 25%), the initial participant recruitment was lower than expected and the study was underpowered.

### Blinding

No blinding was undertaken. GPs and patients were required to make an informed decision for consent so had full disclosure of the intervention and the study design. To determine and compare pre- and post-intervention periods for each participant the intervention status was known.

### Statistical methods

The distribution of demographic characteristics, caring status and living situation was compared between Steps at each time window. Age in years, as a median, was compared using Kruskal-Wallis H-tests, while categorical variables were compared using chi-squared analyses.

Presentations to the ED and admissions to hospital were count data. Person days in the study was the number of participants, multiplied by the number of days in the study during each time window For example Window 1 was the 90 days before baseline data collection, Window 2 was the subsequent 90 days, etc. This was calculated as a total for each GP cluster and time window. In cases where an individual had a hospital admission and/or ED presentation, the length of stay for these events was subtracted from their person-days in the study. The incidence rate of ED presentations and hospital admissions were calculated as the number of events divided by the participant days in the study, multiplied by 1000. Rates were compared between Steps using the STATA ‘iri’ function, which calculates point estimates and confidence intervals for incidence-rate ratios.

To estimate the effect of the intervention, after accounting for differences between the Steps, the OPEN ARCH data were transformed into long format by time window and analysed with multilevel mixed-effects Poisson regression models using the STATA ‘meqrpossion’ function. ED presentations and hospital admissions were analysed using separate models, each of which had three iterations. The first unadjusted model (Model 1) consisted of a dependent variable (e.g. number of ED presentations) and intervention status (i.e. intervention or control) as the independent variable, with random effects for Step, Cluster and individual. This model was then adjusted for time window (Model 2) to determine whether there were any changes across time and then adjusted for demographics (Model 3), to account for the differences in patient characteristics between Steps. These mixed effects models were undertaken using an ‘analysis by treatment allocated” approach, which assumed that the OPEN ARCH intervention had an immediate effect on rates. For participants in Step 1 for example, events during Window 1 were considered as occurring during a control period and events in Window 2 as during an intervention period. As there was likely a delayed benefit of the intervention, the mixed effects modelling was also undertaken using a ‘pragmatic’ approach. In this approach, the first window after the intervention commenced was also coded as a control period. In this case, for Step 1, events during both Window 1 and Window 2 were therefore considered as occurring during the control period and Window 3 represented the first intervention period.

All analyses were undertaken using STATA 14 (StataCorp. 2015. Stata Statistical Software: Release 14. College Station, TX: StataCorp LP) and significance was set at 0.05.

## Results

### Participant flow

**Fig. 3:**
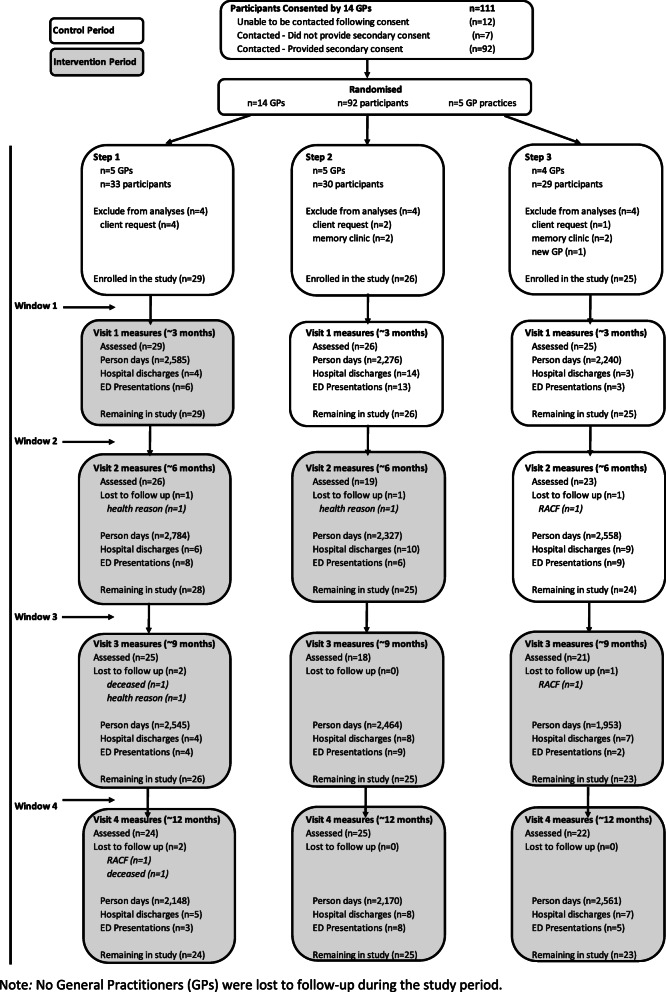
Random selection and flow of participants during the OPEN ARCH study. Note: No General Practitioners (GPs) were lost to follow-up during the study period

General Practitioners within the OPEN ARCH program identified 111 patients for this study. Of these, 12 were unable to be contacted by the OPEN ARCH research team and 7 did not provide secondary consent for the study. A total of 92 participants were recruited across 14 GPs (Fig. 3), of which 7 withdrew consent, 4 commenced a geriatrician-led memory clinic, and 1 participant changed GP, before study commencement, and these were removed from the study. 80 participants commenced the study and entered Window 2, 77 entered Window 3, 74 entered Window 4, and 72 participants completed the study (Fig. 3). Figure 3 details the reason for individual participant attrition and the associated effect on person-days.

### Baseline data

The baseline characteristics of OPEN ARCH participants are described in detail elsewhere [23]. In summary, there were more females (55%) than males, more than half the participants were aged over 80 years (56.3%), and approximately 15% of participants identified as Indigenous. More than half the participants did not have a carer (56.3%) and almost all (92%) received a pension.

Demographic characteristics were collected from participants at baseline only. As such, the caring and living situation of each participant were assumed to have not changed over the duration of the study. There was no significant difference in age, gender, or living situation between steps and this remained true over time (Table 1). A significant difference between steps was noted for Indigenous status (*p* = 0.025), and the presence of a family carer(*p =* 0.02) with significance maintained for each characteristic across time windows (Table 1).

**Table 1:** Characteristics of 80 participants in the OPEN ARCH study, by Step of transition into the intervention and time window between assessments

Measure	Step	**Window**			
		1	2	3	4
**Number (Participants per Window)**	1	29	29	28	26
	2	26	26	25	25
	3	25	25	24	23
**Age (years) Median (IQR)**	1	79 (77–84)	79 (77–84)	79 (77–84)	79 (77–84)
	2	78.5 (74–87)	78.5 (74–87)	78 (74–87)	78 (74–87)
	3	83 (80–86)	83 (80–86)	83.5 (79.5–86)	84 (80–86)
*Kruskal Wallis [Chi, P]*		*[3.04, 0.219]*	*[3.04, 0.219]*	*[2.85, 0.240]*	*[2.99, 0.225]*
**Male Percent (95% CI)**	1	34.5 (17.2–51.8)	34.5 (17.2–51.8)	35.7 (18.0–53.5)	30.8 (13.0–48.5)
	2	53.8 (34.7–73.0)	53.8 (34.7–73.0)	56.0 (36.5–75.5)	56.0 (36.5–75.5)
	3	48.0 (28.4–67.6)	48.0 (28.4–67.6)	45.8 (25.9–65.8)	47.8 (27.4–68.2)
*Chi Square [Chi, P]*		*[2.21, 0.331]*	*[2.21, 0.331]*	*[2.19, 0.334]*	*[3.42, 0.180]*
**Indigenous Percent (95% CI)**	1	20.7 (5.9–35.4)	20.7 (5.9–35.4)	21.4 (6.2–36.6)	19.2 (4.1–34.4)
	2	23.1 (6.9–39.3)	23.1 (6.9–39.3)	24.0 (7.3–40.7)	24.0 (7.3–40.7)
	3	0.0	0.0	0.0	0.0
*Chi Square [Chi, P]*^a^		*[6.48, 0.025]*	*[6.48, 0.025]*	*[6.50, 0.025]*	*[6.06, 0.032]*
**Family Carer Percent (95% CI)**	1	27.6 (11.3–43.9)	27.6 (11.3–43.9)	28.6 (11.8–45.3)	23.1 (6.9–39.3)
	2	46.2 (27.0–65.3)	46.2 (27.0–65.3)	48.0 (28.4–67.6)	48.0 (28.4–67.6)
	3	12.0 (−0.7–24.7)	12.0 (− 0.7–24.7)	8.3 (−2.7–19.4)	8.7 (− 2.8–20.2)
*Chi Square [Chi, P]*^a^		*[12.22, 0.020]*	*[12.22, 0.020]*	*[14.49, 0.006]*	*[14.64, 0.006]*
**Lives with Family Percent (95% CI)**	1	17.2 (3.5–31.0)	17.2 (3.5–31.0)	17.9 (3.7–32.0)	15.4 (1.5–29.3)
	2	23.1 (6.9–39.3)	23.1 (6.9–39.3)	24.0 (7.3–40.7)	24.0 (7.3–40.7)
	3	12.0 (−0.7–24.7)	12.0 (−0.7–24.7)	12.5 (− 0.7–25.7)	13.0 (− 0.7–26.8)
*Chi Square [Chi, P]*^a^		*[2.92, 0.574]*	*[2.92, 0.574]*	*[3.50, 0.471]*	*[3.58, 0.457]*

^a^Fischers Exact due to small cell numbers

In the first time window (Window 1) 11 participants (13.8%) had an ED presentation during the first and 12 (15.0%) a hospital admission.

Table 2 shows the rate of ED presentations in the Intervention period (2.52, 95%CI 2.48–4.48) was comparable to the rate in the Control period (2.80 per 1000, 95%CI 1.99–2.82, IRR = 0.09, 95%CI 0.66–1.27, *p* = 0.254). While the rate of hospital admissions was slightly lower in the intervention period (2.59, 95%CI 1.83–3.55) compared with the control period (3.37, 95%CI 1.78–3.48), the difference was only at trend significance (IRR = 0.77, 95%CI 0.58–1.05, *p* = 0.080).

**Table 2:** Person days, Emergency Department (ED) Presentations and Hospital Admissions expressed as rates per 1000 person days, for participants in the OPEN ARCH study by Step, differences between Intervention and Control Periods analysed as Incident Rate Ratios (IRR)

	Person days	ED Presentations	Hospital Admissions
**Control Period**			
**Step 1**			
Window 1	2585	6	4
**Step 2**			
Window 1	2276	13	14
Window 2	2327	6	10
**Step 3**			
Window 1	2240	3	3
Window 2	2558	9	9
Window 3	1953	2	7
**Total days or events**	**13,939**	**39**	**47**
**Total Rate**		**2.80**	**3.37**
**95% CIs**		**(1.99–3.82)**	**(1.78–3.48)**
**Intervention Period**			
**Step 1**			
Window 2	2784	8	6
Window 3	2545	4	4
Window 4	2148	3	5
**Step 2**			
Window 3	2464	9	8
Window 4	2170	8	8
**Step 3**			
Window 4	2561	5	7
**Total days or events**	**14,672**	**37**	**38**
**Total Rate**		**2.52**	**2.59**
**95% CIs**		**(2.48–4.48)**	**(1.83–3.55)**
**Intervention Period compared to Control Period**			
**Incidence Rate Ratio (IRR)**			
**IRR**		0.90	0.77
**95% CIs**		(0.66–1.27)	(0.58–1.05)
**1 sided p**		0.254	0.040
**2 sided p**		0.508	0.080

Rate = (Events/Person Days) *1000. *95%Cis* 95% Confidence Intervals

Mixed effects modelling using the analysis by treatment allocated approach indicated no effect of the intervention on ED presentations (Model 1– IRR = 0.91, 95%CI 0.56–1.47, *p* = 0.697). This result remained stable after adjusting for time period (Model 2– IRR = 1.35, 95%CI 0.57–3.17, *p* = 0.498) and demographics (Model 3–IRR = 1.17, 95%CI 0.52–2.66, *p* = 0.703) (Table 3). A similar trend was observed for hospital admissions. When all mixed effects analyses were undertaken using a ‘pragmatic approach’, there remained no effect from the intervention (results not tabled).

**Table 3:** Multilevel mixed-effects Poisson regression modelling with Incident Rate Ratios (IRR) for Emergency Department (ED) Presentations and Hospital Admissions unadjusted and adjusted for time period and demographics

	**Model 1**			**Model 2**			**Model 3**		
	IRR	(95% CI)	P	IRR	(95% CI)	P	IRR	(95% CI)	P
**ED Presentations**									
**Intervention Status**	0.91	(0.56–1.47)	0.697	1.35	(0.57–3.17)	0.498	1.17	(0.52–2.66)	0.703
**Time Period**									
**1 (ref)**									
**2**				0.88	(0.46–1.70)	0.709	0.91	(0.47–1.76)	0.787
**3**				0.58	(0.22–1.51)	0.263	0.64	(0.25–1.66)	0.361
**4**				0.60	(0.20–1.79)	0.361	0.69	(0.24–1.97)	0.484
**Indigenous Status**							2.27	(0.63–8.14)	0.208
**Age**							0.97	(0.91–1.04)	0.417
**Gender**							0.74	(0.33–1.63)	0.451
**Hospital Admissions**									
**Intervention Status**	0.93	(0.58–1.47)	0.743	0.62	(0.28–1.40)	0.253	0.57	(0.26–1.27)	0.167
**Time Period**									
**1 (ref)**									
**2**				1.23	(0.67–2.27)	0.499	1.26	(0.68–2.32)	0.457
**3**				1.48	(0.64–3.46)	0.36	1.59	(0.68–3.69)	0.283
**4**				1.90	(0.68–5.34)	0.224	2.07	(0.74–5.75)	0.165
**Indigenous Status**							2.41	(0.53–10.87)	0.252
**Age**							0.97	(0.90–1.05)	0.443
**Gender**							0.80	(0.32–1.99)	0.627

Model 1 – Unadjusted, Model 2 – Adjusted for time period, Model 3 – Adjusted for time period, Indigenous status, age and gender

#### Potentially preventable Hospitalisations

There were 13 hospital admissions identified as PPHs during the study, and these were spread almost evenly between the time windows and across the intervention groups (data not tabled). The most common diagnoses were Chronic Bronchitis (J44.11, *n* = 3), Chronic Obstructive Pulmonary Disease (J44.0, *n =* 3), and Congestive Cardiac Failure (I50.0, *n* = 2). As there were only a small number of PPHs, no further analyses were undertaken.

### Harms

There were no harms reported in this trial.

## Discussion

The OPEN ARCH intervention was designed to integrate health services at the primary-secondary interface in a preventative and comprehensive approach to geriatric care. This study aimed to determine whether the OPEN ARCH intervention influenced the rate of ED presentation or hospital admissions of study participants. The results indicate that the OPEN ARCH intervention did not make a statistically significant difference to the primary outcomes. However, a stabilising of ED presentations and a trend toward lower hospitalisation rates while not statistically significant (at the 5% level) are clinically important findings as functional decline and a related increase in non-preventable hospital use could be expected within this population group over time [1, 3].

Although the stepped wedge RCT has been implemented elsewhere as a robust method of health service evaluation when adequate power is reached [24, 25], the short trial period of the OPEN ARCH study and the low participant numbers were considerable limitations that impacted the capacity for the study to show effect. The malalignment of research requirements and project deliverables is of note here. Fixed-term project funding plus a substantial set-up period eroded the intervention period and compromised the capacity for extensive participant recruitment. These limitations are similar to those reported in the first round of Australian Coordinated Care trials. In these trials, brief project timeframes and limitations in evaluation design compromised the capacity for the intervention to measure benefit to participants [26]. These same constraints were also identified as key factors in the failure of other integrated care programs to show effect [27].

Despite the noted limitations, OPEN ARCH is the only Australian-based program of its kind (i.e. integrating geriatric care at primary-secondary interface) that has evaluated the impact on hospital use via a randomised controlled trial. While other similar Australian integrated care programs have reported a positive impact on ED presentations and hospitalisations (HART reported a 20.8% reduction in ED presentations and a 27.9% reduction in hospital admissions, and Health One Mt Druitt reported a significant difference in ED presentations amongst participants) each of these interventions utilised a pre-post design and neither included a comparator group [13, 14].

As a preventatively focussed program of care, OPEN ARCH sought to intervene early in the trajectory of the participant’s illness and did not include the frequency of ED presentation or hospitalisation as eligibility criteria. As such, only 11 participants (13.8%) had an ED presentation during the first time window and 12 (15.0%) a hospital admission. This contrasts with both HARP and Health One Mt Druitt in which participants having at least three ED presentations in the 12 months prior to program enrolment was an eligibility criterion [13, 14]. The lower numbers of individuals presenting to ED or being admitted in the OPEN ARCH study means that a significant impact on these measures would be difficult to show in the time available and may represent a less vulnerable cohort than HARP or Health One.

Literature suggests that comprehensive evaluation of integrated and community focussed models of care must include both patient-reported outcomes and evaluation measures [27–29]. The study reported here is only one component of the larger OPEN ARCH evaluation in which the patient experience has been explored with positive results, and patient-reported outcome measures of function and quality of life will be examined (18, [30] 31).

## Conclusion

Results indicate that while this study detected no statistically significant different change in ED presentations or hospital separations (discharges), stabilising of ED presentation and hospitalisation rates is a clinically significant finding for older persons with complex needs. However, a longitudinal perspective is required to determine longer-term impact. The complexity of implementing integrated approaches to care must be considered when planning the evaluation of such programs. A multi-faceted approach to the evaluation of integrated care interventions that includes patient-reported outcomes and experience measures is essential to accurately determine the effectiveness of the intervention.

## Supplementary Information

**Additional file 1: Supplementary Table 1** - Number of participants and person days in each step and each cluster, per time window. **Supplementary Table 2** - Number of ED presentations and rate by step and cluster, per time window. **Supplementary Table 3** - Number of Hospital separations and rate by step and cluster, per time window.

## Data Availability

The datasets generated and/or analysed during the current study will be made available on request to the corresponding author with the appropriate ethics and governanceapproval.

## References

[1] McPake B, Mahal A (2017). Addressing the needs of an ageing population in the health system: the Australian case. Health Syst Reform.

[2] Katterl R, Anikeeva O, Butler C, Brown L, Smith B, Bywood P (2012). Potentially avoidable hospitalisations in Australia: Causes for hospitalisations and primary health care interventions. [Internet]. Primary Health Care Research & Information Service: PHCRIS Policy Issue Review. Primary.

[3] Beswick AD, Rees K, Dieppe P, Avis S, Gooberman-Hill R, Horwood J, Ebrahim S (2008). Complex interventions to improve physical function and maintain independent living in elderly people: a systematic review and meta-analysis. Lancet..

[4] Agency for Clinical Innovation (2014). Building Partnerships: A framework for integrating care for older people with complex health needs.

[5] Tieman J, Mitchell G, Shelby-James T, Currow D, Fazekas B, O’Doherty L, Hegarty M, Erikkson L, Brown R, Reid-Orr D (2007). Integration, coordination and multidisciplinary care: what can these approaches offer to Australian primary health care?. Aust J Prim Health.

[6] Kuipers P, Kendall E, Ehrlich C, Amsters, Kendall M, Kuipers K, et al. Complexity and healthcare: health practitioner workforce services, roles, skills and training to respond to patients with complex needs. [Internet]. Clin Educ Train Queensland. 2011; Available at: https://www.health.qld.gov.au/__data/assets/pdf_file/0027/150768/complexcarefull1.pdf [cited on 2021 Feb 2].

[7] Pond D. C., Regan C. Improving the delivery of primary care for older people. [Internet]. Med J Aust. 2019;21(2):60–62.e1.doi: 10.5694/mja2.50236 10.5694/mja2.50236 PMC6852290 31206179

[8] Mitchell GK, Burridge L, Zhang J, Donald M, Scott IA, Dart J, Jackson CL (2015). Systematic review of integrated models of health care delivered at the primary-secondary interface: how effective is it and what determines effectiveness?. Aust J Prim Health.

[9] Integrated care for older people: guidelines on community-level interventions to manage declines in intrinsic capacity. [Internet]. Geneva: World Health Organization. 2017. Licence: CC BY-NC-SA 3.0 IGO. Accessed at: https://apps.who.int/iris/bitstream/handle/10665/258981/9789241550109-eng.pdf;jsessionid=E5EC73932300AB013FDFFF43EF72E02E?sequence=1 [cited on 2021 Feb 2]. 29608259

[10] Valentijn PP, Schepman SM, Opheij W, Bruijnzeels MA (2013). Understanding integrated care: a comprehensive coneptual framework based on the intgrative functions of primary care. [Internet]. Int J Integr Care.

[11] Carswell P (2015). Te Whiringa Ora: person-centred and integrated care in the Eastern Bay of Plenty, New Zealand. [Internet] Int J Integrated Care.

[12] Wodchis WP, Dixon A, Anderson GM, Goodwin N. Integrating care for older people with complex needs; key insignts and lessons from a seven-country cross-case analysis. [Internet]. Int J Integr Care. 2015;15 Available at https://www.ijic.org/articles/10.5334/ijic.2249/ [cited on 2021 Feb 02]. 10.5334/ijic.2249 PMC4628509 26528096

[13] Bird SR, Kurowski W, Dickman GK, Kronborg I (2007). Integrated care facilitation for older patients with complex health care needs reduces hospital demand. Aust Health Rev.

[14] McNab J, Mallitt K, Gillespie J. Report of the evaluation of HealthOne Mount Druitt. [Internet]. Menzies Centre Health Policy. 2013; Available at https://ses.library.usyd.edu.au/bitstream/handle/2123/8988/HOMDevaloct13.pdf [cited on 2021 Feb 2].

[15] State of Queensland (2019). Integrated Care Innovation Fund.

[16] Mann J, Quigley R, Harvey D, Tait M, Williams G, Strivens E. OPEN ARCH: Integrated care at the primary-secondary interface for the community-dwelling older person with complex needs. [Internet]. Aust J Prim Health. 2020;26(2) Available at: https://doi: 10.1071/PY19184[cited on 2021 Feb 2]. 10.1071/PY19184 32290951

[17] Kinchin I, Jacups S, Mann J, Quigley R, Harvey D, Doran CM, et al. Efficacy and cost-effectiveness of a community -based model of care for older patients with complex needs: a study protocol for a multicentre randomised controlled trial using a stepped wedge cluster design. [Internet]. Trials. 2018;19(668) Available at: https://doi.org/10.1186/s13063-018-3038-0 [cited on 2021 Feb 2]. 10.1186/s13063-018-3038-0 PMC6280415 30514378

[18] Brown CA, Lilford RJ (2006). The stepped wedge trial design: a systematic review. BMC Med Res Methodol.

[19] Queensland Government (2018). Cairns and Hinterland Hospital and Health Service Clinical Service Plan 2018–2022. [Internet]. State of Queensland, Cairns and Hinterland Hospital and Health Service.

[20] Queensland Health Casemix and Clinical Costing, Supporting Services. [Internet] 2010. Available at: https://www.health.qld.gov.au/redcliffe/work/ss-casemix [cited on 2021 Feb 2].

[21] World Health Organization. International Statistical Classification of Diseases and Related Health Problems 10th revision, 2nd ed. Available at: https://apps.who.int/iris/handle/10665/42980 [cited on 2021 Feb 2].

[22] Australian Institute of Health and Welfare (2018). National Healthcare Agreement: PI 18–Selected potentially preventable hospitalisations. [Internet]. AIHW.

[23] Mann J, Thompson F, Quigley R, McDermott R, Devine S, Strivens E. Beyond multimorbidity: primary care and the older person with complex needs. [Internet]. Aust J Prim Health. 2021; Available at:https://doi.org/10.1071/PY20125 [cited on 2021 Feb 12]. 10.1071/PY20125 33535025

[24] Snooks H, Bailey-Jones K, Russell IT, et al. Effects & costs of implementing predictive risk stratification in primary care: randomised stepped wedge trial. BMJ Qual Saf. 2018. 10.1136/bmjqs-2018-007976 (more than 260 000 participant. 10.1136/bmjqs-2018-007976 PMC6820297 30397078

[25] Esterman AJ, Ben-Tovim DI (2002). The Australian coordinated care trials: success of failure? The second round of trials may provide more answers. Med J Aust.

[26] Kumpunen S., Edwards N., Georghiou T., Hughes. Evaluating integrated care. Why are evaluations not producing the results we expect? Briefing Paper November 2019. [Internet] Nuffield Trust. Available at https://www.nuffieldtrust.org.uk/files/2019-11/the-challenges-of-evaluating-integrated-care-briefing-3.pdf [cited on 2021 Feb 2].

[27] Stoop A, Lette M, Ambugo EA, Gadsby EW, Goodwin N, MacInnes J (2020). Improving Person-Centredness in Integrated Care for Older People: Experiences from Thirteen Integrated Care Sites in Europe. [Internet]. Int J Integr Care.

[28] Quigley R, Russell S, Harvey D, Mann J. OPEN ARCH Integrated care model: experiences of older Australian and their carers. Aust J Prim Health. 2021;27(3):236–42. 10.1071/PY20203. 10.1071/PY20203 33653509

[29] Herzog A, Gaertner B, Scheidt-Nave C, Holzhausen M. ‘We can only do what we have the means for’ General Practitioners’ views of primary care for older people with complex health problems. [Internet]. BMC Fam Pract. 2015;16(35). 10.1186/s1287-015-0249-2. 10.1186/s12875-015-0249-2 PMC4371843 25886960

[30] Mitchell GK, Burridge L, Zhang J, Donald M, Scott IA, Dart J, Jackson CL (2019). Systematic review of integrated models of health care delivered at the primary-secondary interface: how effective is it and what determines effectiveness. [Internet]. Aust J Prim Health.

