# Supplementary material for: Impact of an integrated community-based model of care for older people with complex conditions on hospital emergency presentations and admissions: a step-wedged cluster randomized trial
Source: BMC Health Serv Res. 2021 Jul 16;21:701. doi: 10.1186/s12913-021-06668-x (PMC8285878; doi:10.1186/s12913-021-06668-x)
Supplement: Supplementary file 1 — Additional file 1: Supplementary Table 1 - Number of participants and person days in each step and each cluster, per time window. Supplementary Table 2 - Number of ED presentations and rate by step and cluster, per time window. Supplementary Table 3 - Number of Hospital separations and rate by step and cluster, per time window. [file 12913_2021_6668_MOESM1_ESM.docx]

**Supplementary Table 1 - Number of participants and person days in each step and each cluster, per time window**


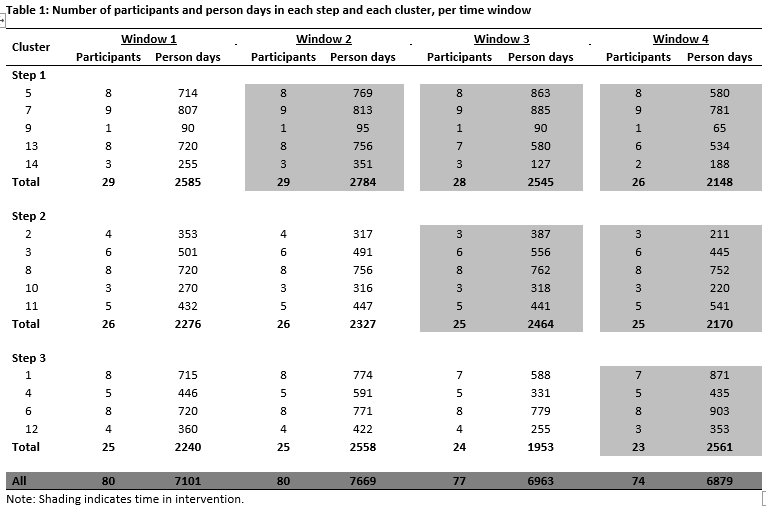


**Supplementary Table 2 - Number of ED presentations and rate by step and cluster, per time window**

**Supplementary Table 3 - Number of Hospital separations and rate by step and cluster, per time window**
